# Anthocyanin Induction by Drought Stress in the Calyx of Roselle Cultivars

**DOI:** 10.3390/molecules25071555

**Published:** 2020-03-28

**Authors:** Jeny Hinojosa-Gómez, César San Martín-Hernández, José B. Heredia, Josefina León-Félix, Tomás Osuna-Enciso, María D. Muy-Rangel

**Affiliations:** 1 Centro de Investigación en Alimentación y Desarrollo, A.C. (CIAD) Coordinación Culiacán, Carretera Eldorado km 5.5, Campo el Diez, C.P. 80110 Culiacán Rosales, Sinaloa, Mexico; jeny.hinojosa@estudiantes.ciad.mx (J.H.-G.); jbheredia@ciad.mx (J.B.H.); ljosefina@ciad.mx (J.L.-F.); osuna@ciad.mx (T.O.-E.); 2 Colegio de Postgraduados, Carretera México-Texcoco km 36.5. Montecillo, C.P. 56230 Texcoco, Estado de México, Mexico; sanmartin.cesar@colpos.mx

**Keywords:** *Hibiscus sabdariffa*, anthocyanins, colour, drought stress, greenhouse, UPLC-MS/MS analysis

## Abstract

Abiotic factors can alter the chemical profile of crops and the number of compounds they contain. In this study, the anthocyanin and anthocyanidin contents, determined by ultra-performance liquid chromatography (UPLC-MS/MS), and the colour attributes of the calyces of three cultivars of *Hibiscus sabdariffa* subjected to three water stress regimes during the stage of physiological maturity were investigated. The total anthocyanin content in calyx increased relative to the control content under a 65% moisture irrigation regime. Among the cultivars, UAN16-2 showed the greatest increases in the contents of cyanidin, delphinidin 3-*O*-glucoside, cyanidin 3-*O*-glucoside, and cyanidin 3-*O*-sambubioside. The content of cyanidin 3-*O*-sambubioside showed the greatest increase, increasing by 55% relative to the control level. The contents of these compounds are correlated with colour attributes such as luminosity. Water stress under the 33% moisture condition during plant development led to decreased anthocyanin contents in all of the roselle cultivars.

## 1. Introduction

*Hibiscus sabdariffa L*., a plant that grows in tropical and subtropical regions, is of considerable medicinal and economic value worldwide. It is valued in the food and nutraceutical industries as a source of minerals and fiber and thanks to its high contents of phenolic compounds, especially anthocyanins [1,2,3,4,5].

The colour of the calyx of this plant, known as roselle, varies from light to intense red and is related to its phytochemical content. Dark calyces have higher anthocyanin contents than do light calyces [6]. Delphinidin-3-sambubioside and cyanidine-3-sambubioside have been reported to be the two main anthocyanins in roselle and delphinidin-3-glucoside and cyanidin-3-glucoside are found in smaller amounts [7]. Approximately 85% of anthocyanins are attributable to delphinidin-3 sambubioside, which is considered the main source of the antioxidant capacity in roselle [8]. The conditions during crop production have strong effects on the concentrations of the main phenols in the calyx. The anthocyanin concentration of some varieties can differ by twofold owing to variation in environmental conditions and soil type at the production site [9]. Therefore, these factors should be considered when defining the chemical composition of a cultivar.

The production of phytochemicals in fruits varies with the stage of development, stress level, defense mechanisms of the plant, genotype, and genotype*environment interactions [10,11]. It has been reported that water stress induces oxidative stress as a result of the formation of free radicals; to counteract the damage, the plant generates a signaling cascade, activating defense mechanisms that favor the production of secondary metabolites [12,13,14,15].

Although water stress adversely impacts the growth and development of plants [12], several studies have shown that low levels of water stress can induce the production of secondary metabolites, such as flavonoids, that function to regulate metabolic processes that serve in plant defense [16,17,18,19]. In addition, they serve as antioxidant compounds that reduce the turgor loss of the cell membrane, decreasing the cell lesions associated with stress [20].

The production environment of roselle plants has a strong effect on the concentrations of the main phenols present in the calyx. Variation of up to 70% in the contents of total soluble phenolic compounds and anthocyanins according to the production site has been reported [9]. In addition, the authors of [21] demonstrated that drought stress during roselle cultivation increases the anthocyanin content of the calyx by up to 28%. However, it is unknown whether this effect on plant quality persists at different levels of drought and in different cultivars in commercial production.

The hypothesis evaluated in the present study is that moderate drought stress during roselle development modifies the contents of secondary metabolites and improves the contents of anthocyanins and some minerals and nutritional compounds in the calyx. The objective of this study was to evaluate the effect of water stress on anthocyanin content, which determines calyx colour, in three cultivars of roselle cultivated under three irrigation regimes under hydroponic and greenhouse conditions.

## 2. Results and Discussion

### 2.1. Anthocyanins Produced Under Water Stress

The *H. sabdariffa* calyx contained two anthocyanidins (cyanidin and delphinidin) and four anthocyanins (cyanidin 3-*O*-glucoside, cyanidin 3-sambubioside, delphinidin 3-*O*-glucoside, and delphinidin 3-sambubioside), as identified by ultra-performance liquid chromatography (UPLC) (Table 1).

**Figure 1:**
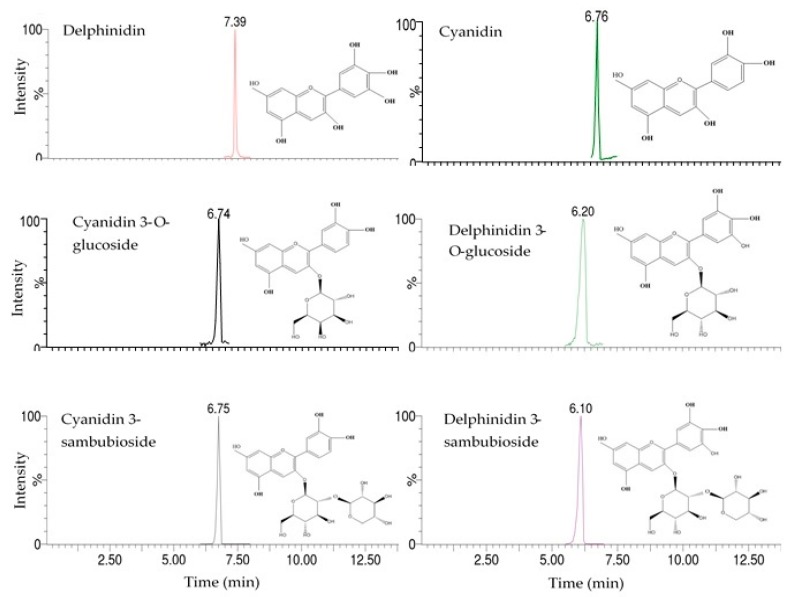
Chromatograms of anthocyanins and anthocyanidins determined in roselle calyx extracts.

**Figure 2:**
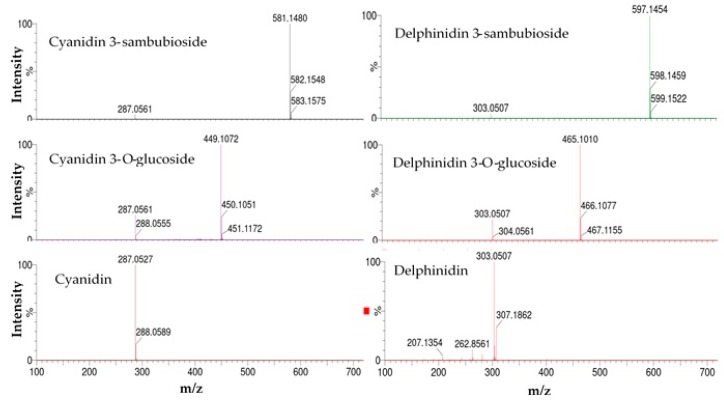
Mass spectra with a collision energy of 10 V for the identification of anthocyanins and anthocyanidins.

Among the identified compounds, cyanidin 3-*O*-glucoside and delphinidin were confirmed based on comparison with authentic standards. The remaining compounds were identified based on their retention time (RT) values, UV spectra, molecular weight, and molecular ion information (Figure 1 and Figure 2) [22].

In all three roselle cultivars, the highest anthocyanin content was achieved in the plants treated with the irrigation regime of 65% (IR2) (Table 2). This result is consistent with the observation that the accumulation of anthocyanins in plants is regulated by various environmental stresses, such as drought, UV, injury, pathogen attack, and nutrient deficiency [23,24].

The highest contents of total anthocyanins were observed in cultivars UAN16-2 and Cruza Negra at IR2 (1066 and 1660 mg EC3G 100 g^−1^ db, respectively) (Table 2). However, water stress under this irrigation regime (65% moisture) induced higher contents of total anthocyanins in all three cultivars of roselle, with content increases of 23%, 72%, and 92% for the cultivars Cruza Negra, 4Q4, and UAN16-2, respectively, relative to the control contents (under 100% moisture). Under extreme water stress, anthocyanin contents were reduced in UAN16-2 and Cruza Negra. In plants, stress can inhibit the activity of the enzyme ascorbate peroxidase (APX), which functions to detoxify hydrogen peroxide by removing water from the vacuoles. As a result, 75% of the peroxide produced in the chloroplast migrates to the cytoplasm and is transported to the vacuole by means of aquaporins; a lack of APX activity stimulates the production of anthocyanins to replace APX function and prevent cell damage [25,26]. Studies on the effects of drought and osmotic stress on polyphenols in different species indicate that polyphenol contents can increase or decrease depending on plant species and the type and intensity of stress [27], as observed in the present study. Increases have been reported in the total phenolic content and in betalains in red beets under drought-induced stress [28]. Furthermore, the concentration of flavonoids was shown to generally decrease in five cultivars of cherry tomatoes under water stress at 50% substrate humidity [29].

**Figure 3:**
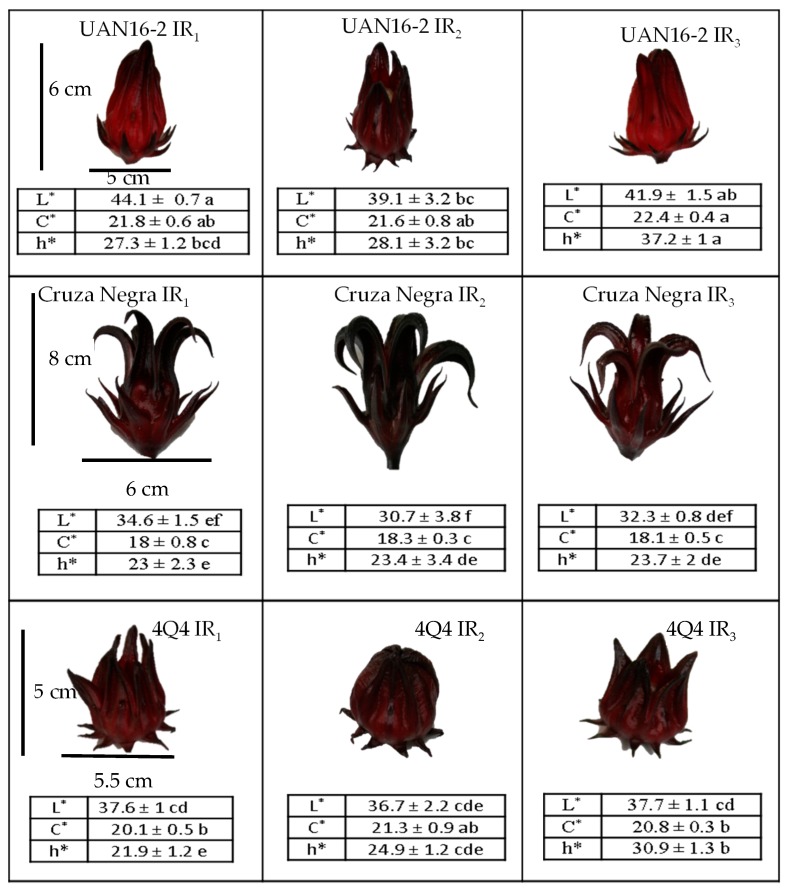
Color parameters and calyx appearance of different roselle genotypes in three irrigation regimes (IR1 = 100%, IR2 = 65%, and IR3 = 33% humidity). Means with different letters within columns and rows per response variable show significant differences according to the Tukey test (*p* ≤ 0.05).

Under water stress (IR2), the cultivar UAN16-2 had the highest contents of cyanidin 3-*O*-sambubioside and delphinidin 3-*O*-sambubioside among the cultivars, with increments of 54% and 43%, respectively, relative to the control contents (under IR1). This suggests that the stress favored the accumulation of anthocyanins with the *O*-sambubioside glucoside, possibly to store compounds with a high number of sugars as energy reserves. These results are interesting because UAN16-2 produces calyces of low-saturation red tones under normal conditions; however, under drought exposure, the red colour of the calyces becomes saturated (Figure 3), and the calyces become similar in colour and pigment content to those of 4Q4.

### 2.2. Roselle Calyx Colour 

In the colour analysis (Figure 3), the control calyces showed significant differences (*p* ≤ 0.05) in luminosity (L), chromaticity, and hue angle (° Hue) among the cultivars, with Cruza Negra showing the lowest hue (° Hue = 23), corresponding to a dark red colour. The colour of the calyces reflected the content of anthocyanins (which are responsible for the red colour). However, the stress conditions yielded notable changes from control conditions only in the luminosity of the roselle calyx in the three cultivars, which was lower under water stress than under 100% moisture (Table 2). In general, the colour of the roselle calyx is very varied and can present values of luminosity between 29 and 49, chromaticity between 18 and 27, and hue angle between 13 and 28 [2], which correspond to calyces of light red, deep red, and dark brown red. However, each variety of roselle has market value owing to its visual quality, size, anthocyanin content, and chemical characteristics such as acidity and aroma [6].

## 3. Materials and Methods 

### 3.1. Field Experiment

**Figure 4:**
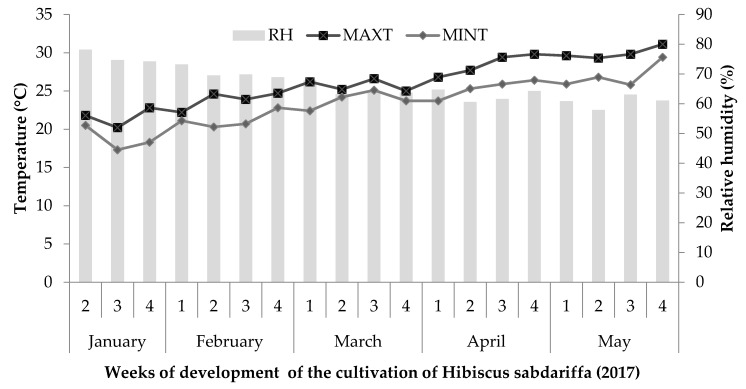
Weekly average of relative humidity (RH) and maximum (MAXT) and minimum temperatures (MINT) during the cultivation of roselle.

The experiment was conducted in a greenhouse at the Food and Development Research Center (CIAD), A.C., Culiacán Unit, located in Sinaloa, Mexico (28 m altitude, 24°44’02 "N and 107°27’16" W), from January to May 2017. Three cultivars of *Hibiscus sabdariffa* L. (Cruza Negra, UAN16-2 and 4Q4) provided by the Autonomous University of Nayarit, Mexico, were studied under hydroponic conditions in greenhouses. To monitor the environmental conditions, a Hobo Data Logger was placed within the canopy of the plants to record daily temperature and relative humidity (Figure 4).

The seeds were planted in germination trays of 120 cavities in a mixture of peat moss/agrolite (70:30 *v*/*v*). At 14 days after germination, the seedlings were transplanted in a greenhouse. Each seedling was placed in a polyethylene bag containing 13 L of a previously characterized potting mixture (60% fluvisol soil and 40% coconut fiber) (Table 3) with a 0.50 m planting distance and 1.0 m between rows.

**Figure 5:**
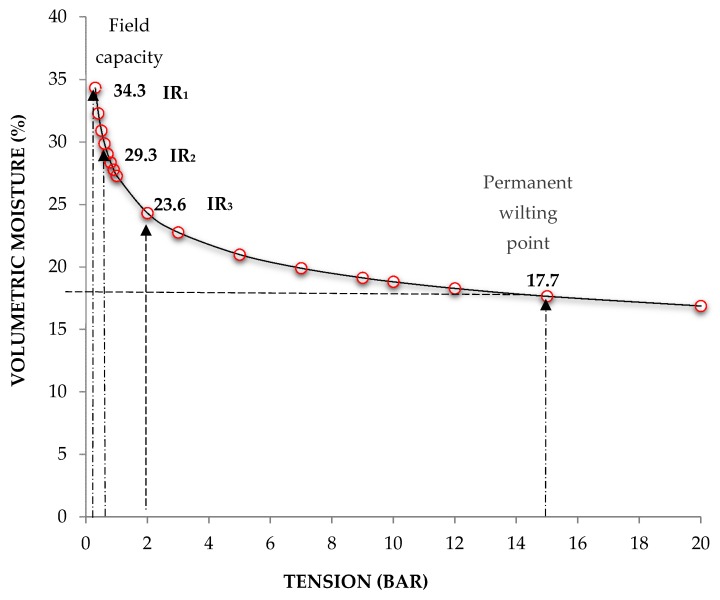
Water retention curve of the substrate.

A two-factor completely randomized design was used, with three levels per factor: factor 1, cultivar (Cruza Negra, UAN16-2 and 4Q4); and factor 2, substrate moisture level (100%, 65%, and 33%). There were three treatments in total, with each unit represented by five plants (one of which was used to monitor substrate moisture) and three experimental replicates per treatment; in addition, there were 56 board plants. To obtain the moisture retention curve of the soil mixture, three samples were taken, pre-saturated, and then subjected to different stresses by the pressure membrane method, applying the principle of hydrostatic equilibrium [30]. Then, on the basis of the curve, three irrigation regimes (IR) were selected: 100% (343 mL L^−1^ soil mixture) humidity at field capacity (FC) (IR1: irrigation regime without deficit in available soil moisture for the plant), 65% of the FC moisture required (293 mL L^−1^) (IR2: irrigation regime with moderate soil moisture deficit), and 33% of the FC required (236 mL L^−1^) (IR3: irrigation level with high soil moisture deficit), all with a moisture range of ± 5% (Figure 5).

Steiner solution [31] with an electrical conductivity of 2 dS m^−1^ and pH at 5.8 was supplied through drip irrigation eight times a day for 5 min using 4 L h^−1^ drippers during the first 90 days of cultivation. From calyx development until harvest (168 days after sowing), the moisture treatments were applied (varying the irrigation dose), and the moisture level in the substrate was continually monitored by the gravimetric method.

The plants were harvested at the commercial maturity stage (at the opening of the capsule, which exposes the seeds), and the calyces were separated manually from the capsules. The calyces were frozen at −20 °C, lyophilized, and stored at −60 °C.

### 3.2. Identification and Quantification of Anthocyanins

The extraction of anthocyanins from the roselle calyces was performed according to [32] with slight modifications. One milligram of lyophilized calyx was weighed, and 10 mL of methanol acidified with 1% HCl was added; the solution was then refrigerated at 4 °C overnight. Subsequently, the solution was filtered with Whatman No. 1 filter paper in a vacuum pump, and 10 mL of methanol acidified with 1% HCl was added to wash the anthocyanin residues. The sample was then evaporated to dryness in a rotary evaporator at 45 rpm at 30 °C, and then re-dissolved in 10 mL of 0.01% HCl (Milli-Q water). To purify the anthocyanins, 5 mL of the extract was added to a separatory funnel and stirred with 10 mL of petroleum ether to collect the colored phase. The sample was again added to the separatory funnel, 10 mL of ethyl acetate was added, the mixture was stirred, and the colored phase was collected. One milliliter of the extract was absorbed in a Sep-Pak C-18 cartridge (Waters Assoc., Milford, MA, USA) previously activated with 1 mL of methanol and 1 mL of 0.01% HCl. The absorbed extract was discarded; then, 2 mL of 0.01% aqueous HCl was filtered and discarded, and the extract was filtered with 4 mL of methanol acidified with 0.01% HCl (Milli-Q water) and the extract was evaporated. After drying in a rotary evaporator at 35 °C, the extract was re-dissolved in 1 mL of Milli-Q water acidified with 0.01% HCl. Finally, the extract was filtered on a nylon filter with a diameter of 13 mm and a particle size of 0.2 μm (Agilent Technologies), and stored in a dark vial at −20 °C for further analysis [33].

Anthocyanidins and anthocyanins (cyanidin, delphinidin, cyanidin-3-*O*-glucoside, cyanidin 3-sambubioside, delphinidin 3-*O*-glucoside, delphinidin 3-sambubioside) were identified and quantified via ultra-performance liquid chromatography (UPLC-MS/MS) Acquity class H (Waters) instrument coupled to a mass analyzer G2-XS Qtof (quadrupole and time-of-flight) using a procedure modified from [34]. Briefly, compounds were separated on a C18 column (Acquity BEH, 1.7 µm, 2.1 mm × 50 mm, Waters, 186,002,350) maintained at room temperature. Mobile phase A was a 99:1 *v*/*v* formic acid/water mixture, and mobile phase B was 100% acetonitrile. The injection volume was 1 μL. The mobile phase gradient is summarized in Table 4.

Electrospray ionization (ESI) was used. [M]^−^ is given as the identity of the molecular ion instead of [M + H]^−^ because, in acidic conditions, there are anthocyanins, such as flavylium cations [35], with a collision energy of 10 V. The electrospray capillary voltage was 1.5 kV, the source temperature was 150 °C, the desolvation temperature was 350 °C, the gas (nitrogen) was provided at a flow rate of 500 L h^−1^, the cone voltage was 40 V, and the collision gas was 20 V. The compounds were identified based on the literature [22] and quantified based on curves of HPLC-grade analytical standards of cyanidin 3-*O*-glucoside (CAS No. 7084-24-4) and delphinidin (CAS No. 528-53-0), with a degree of purity ≥95% for both (Sigma-Aldrich, USA). On the basis of this methodology, the analysis method is considered reliable.

### 3.3. Calibration Curves

Each of the standards (1 g) was diluted in 25 ml of UPLC grade methanol to reach a final concentration of 38,000 ng mL^−1^ (considering 95% purity). Stock solution of the two analytes was diluted to appropriate concentrations (25 dilutions from 0 to 38,000 ng mL^−1^ with proportional increases of 253.3 ng mL^−1^) For each calibration curve, eight of the previous concentrations were used (63.33, 253.33, 760, 1520, 2026.67, 5066.67, 12,666.67, and 22,800 ng mL^−1^), and each point was measured in triplicate for the construction of calibration curves. The calibration curves were constructed by plotting the peak area (extracted ion chromatograms (EIC) signal of MS) versus the concentration of each analyte. For each point of the curve, three injections of the indicated concentration were made.

### 3.4. Colour Analysis 

The colour attributes of the roselle calyx were obtained in the CIELCh colour space using a spectrophotometer (CM-700d; Konica Minolta, Ramsey, New Jersey, USA). The luminosity (L), hue angle (Hue = arc tan b/a), and chromaticity (Chroma = √[a^2^ + b^2^]) were obtained with OnColor QC version 5 (CyberChrome, Stone Ridge, NY, USA) [2].

### 3.5. Statistical Analysis

A completely randomized factorial design was employed with two factors, each with three levels: soil moisture (100%, 65%, and 33%) and cultivar (Cruza Negra, UAN16-2 and 4Q4). The data were analyzed by analysis of variance (ANOVA), and the means were compared with the Tukey test (*p* ≤ 0.05) with Minitab 17 software.

## 4. Conclusions

It is possible to increase the anthocyanin content in the roselle calyx by modifying agricultural practices, for example, by applying decreased irrigation during the stage of physiological maturity of the fruit under greenhouse conditions. The present study found that a moderate water stress irrigation regime (65% moisture) achieved an increase in anthocyanins, and that UAN16-2 was the cultivar with the best response to water stress, exhibiting significant changes in pigment synthesis. Extreme water stress (33% moisture) affected the concentrations of calyx pigments. Anthocyanin content was correlated with the colour properties of the roselle calyces. 

## Figures and Tables

**Table 1:** Compounds identified in *H. sabdariffa* extracts. RT, retention time.

RT (min)	Analyte	Formula	Exact Mass *m*/*z*	Scan Mode	Fragments
6.76	Cyanidin	C_15_H_11_O_6_^+^	287.05	[M]^+^	287.05, 288.05
7.39	Delphinidin	C_15_H_11_O_7_	303.05	[M]^+^	207.13, 262.85, 303.05
6.74	Cyanidin 3-*O*-glucoside	C_21_H_21_O_11_^+^	449.1	[M]^+^	287.05, 288.05, 449.1
6.75	Cyanidin 3-sambubioside	C_26_H_29_O_15_^+^	580.14	[M + H]^+^	287.05, 581.14, 582.15
6.20	Delphinidin 3-*O*-glucoside	C_21_H_21_O_12_^+^	465.1	[M]^+^	303.05, 304.05, 465.10
6.10	Delphinidin 3-sambubioside	C_26_H_29_O_16_^+^	596.14	[M + H]^+^	303.05, 597.14, 598.14

**Table 2:** Anthocyanins and anthocyanidins (mg EC3G 100 g^−1^ db) of the different genotypes of roselle calyces at three irrigation regimes of IR1, IR2, and IR3 (humidity content of 100%, 65%, and 33%, respectively) (mean ± SD).

Cultivar/ Regimen		Cyanidin	Delphinidin	Cyanidin 3-*O*-glucoside	Cyanidin 3-Sambubioside	Delphinidin 3-*O*-Glucoside	Delphinidin 3-Sambubioside	Total Anthocyanins
UAN16-2	IR_1_	40.4 ± 9.5 de	15.9 ± 2.9ef	8.6 ± 1.8de	184.3 ±13.2de	20.6 ± 1.7cd	285.3 ± 9.9f	555.3 ± 33.2e
	IR_2_	71.9 ± 11 bc	28.9 ± 2.7d	16.5 ± 0.1bc	405 ± 11.1b	39.8 ± 4.8a	504.2 ±15cd	1066.3 ± 44.5c
	IR_3_	21.6 ± 2.4e	4.3 ± 0.8f	6.3 ± 1.5e	98.6 ± 3.7f	12.2 ± 1.2d	134.1 ± 13.1g	277.2 ± 22.7f
4Q4	IR_1_	35.9 ± 6.8de	11.3 ± 7.5c	4.6 ± 0.4e	148.4 ± 13.1e	23.2 ± 2.9c	325.3 ± 8.8ef	547.2 ± 7.5e
	IR_2_	55.9 ± 4cd	23.1 ± 2.4ef	12.1 ± 2cd	280.3 ± 11c	34.9 ± 3.2ab	532.6 ± 14.9c	938.9 ± 7d
	IR_3_	42.4 ± 2.5de	8.4 ± 1.1f	7.6 ± 1de	211.8 ± 24.2d	23.2 ± 2.3c	328.1 ± 7.1e	621.5 ± 36e
Cruza Negra	IR_1_	76.3 ± 2.4bc	78.9 ± 1.5b	17.3 ± 1.7b	507.7 ± 1.4a	38.1 ± 2.6 ab	615.9 ± 8.3b	1350.1 ± 10.1b
	IR_2_	104.3 ± 2.4a	95.4 ± 1.5a	23.2 ± 1.7a	540 ± 1.4a	44 ± 2.6a	853.4 ± 8.3a	1660.3 ± 1.1a
	IR_3_	92.1 ± 1.2bc	45.4 ± 7.5c	9.1 ± 1.3de	373.5 ± 5.1b	27.4 ± 1.4bc	488.1 ± 7.4d	1019.8 ± 6.4cd

In each column, different letters mean significant differences between the samples according to the Tukey test (*p* ≤ 0.05).

**Table 3:** Physicochemical profile of the substrate.

Characteristic	Value
pH	6.44
Electrical conductivity (dS m^−1^)	1.14
Sodium (mg kg^−1^)	528.99
Potassium (mg kg^−1^)	801.28
Calcium (mg kg^−1^)	5025.65
Magnesium (mg kg^-1^)	5025.65
Iron (mg kg^−1^)	990.09
Manganese (mg kg^−1^)	48.86
Zinc (mg kg^−1^)	6.7
Copper (mg kg^-1^)	2.68
Cationic exchange capacity (CEC) (meq 100g^−1^)	37.58
Phosphorus (mg kg^−1^)	89.95
Nitrates (mg kg^−1^)	104.69

**Table 4:** Applied ultra-performance liquid chromatography (UPLC) gradient parameters for the elution of anthocyanins.

Time (min)	Solvent A (%)	Solvent B (%)	Flow rate (μL/min)
0	100	0	400
5	91	9	400
7	85	15	400
8	0	100	400
10	0	100	400
10.1	100	0	400
13	100	0	400
